# Genome sequence of a measles virus strain with a novel loss of stop codon mutation in the phosphoprotein gene

**DOI:** 10.1128/MRA.00833-23

**Published:** 2023-12-01

**Authors:** Vanessa Zubach, Helene Schulz, Kihun Kim, Darian Hole, Alberto Severini, Joanne Hiebert

**Affiliations:** 1 Viral Exanthemata and STD Section, National Microbiology Laboratory, Public Health Agency of Canada, JC Wilt infectious Diseases Research Center, Winnipeg, Manitoba, Canada; 2 Department of Microbiology, University of Manitoba, Winnipeg, Manitoba, Canada; 3 Computational and Operational Genomics Section, National Microbiology Laboratory, Public Health Agency of Canada, Winnipeg, Manitoba, Canada; 4 Department of Medical Microbiology and Infectious Diseases, Faculty of Health Sciences, University of Manitoba, Winnipeg, Manitoba, Canada; DOE Joint Genome Institute, Berkeley, California, USA

**Keywords:** measles virus, novel phosphoprotein, NGS

## Abstract

Measles virus genotype B3 coding-complete genome sequence from a 2019 case showed a novel mutation in the phosphoprotein (P) gene that abrogates the established stop codon. A downstream stop codon has been identified, resulting in a putative P that would be 19 amino acids longer than wild type.

## ANNOUNCEMENT

**Fig 1:**
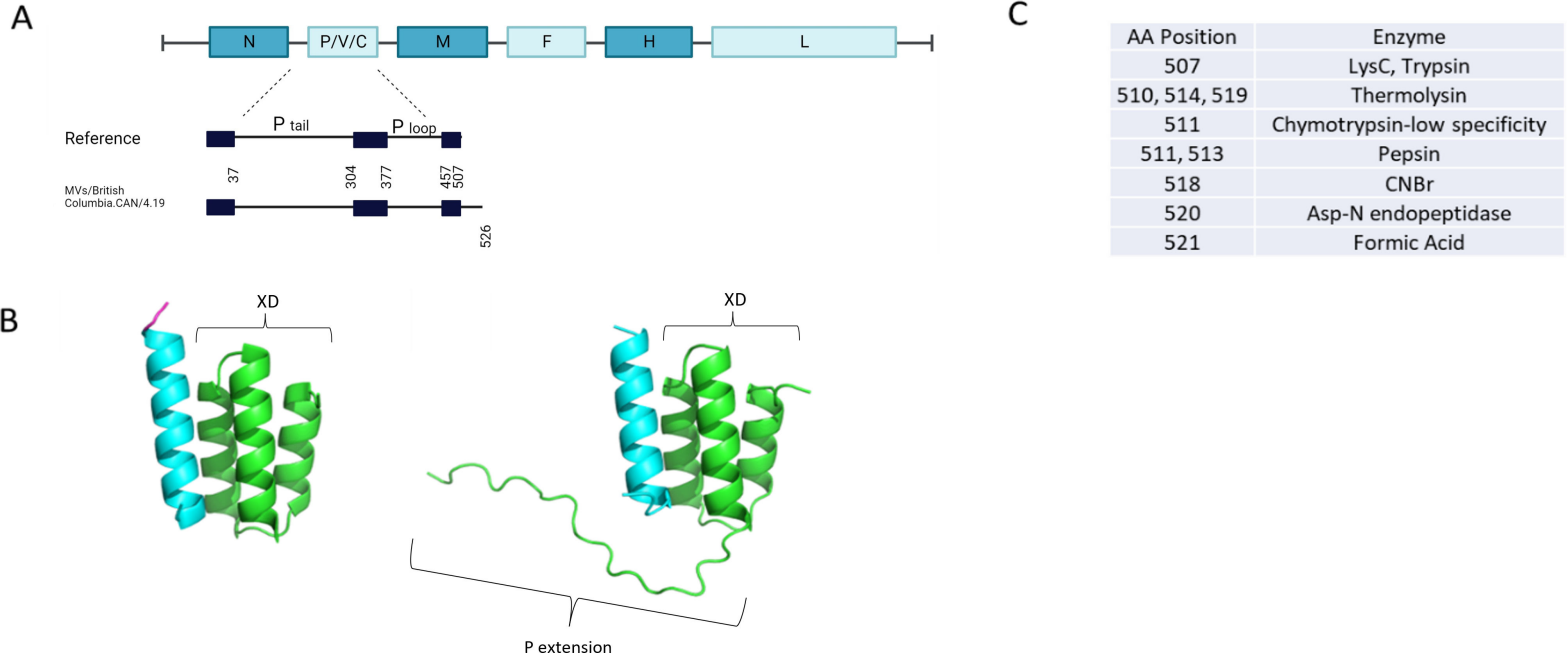
(A) Map of the MeV genome with expansion of the linear protein structure of the wild-type P and the novel sequence. The boxes represent the regions with an ordered P structure. (**B**) Model of the interaction between the N-tail (cyan) and the wild-type (left) and extended (right) XD domain of P (green). (**C**) Potential cleavage sites of the extended P sequence predicted by PeptideCutter.

Measles virus (MeV; genus *Morbillivirus*, family *Paramyxoviridae*) is recognized by the rash it causes (1). Humans are the only hosts. The MeV negative strand RNA genome is typically 15,894 nucleotides long (2) and encodes eight proteins (Fig. 1A). An MeV coding-complete genome sequence [MVs/British Columbia.CAN/4.19 (B3)] with a novel phosphoprotein (P) gene mutation was obtained from a urine specimen and collected from a measles case occurring in 2019, as part of the Canadian national measles surveillance program (3).

Total nucleic acids from clinical specimens were extracted on the MagNA Pure 96 using the DNA and Viral NA large volume kit (catalog 06374891001, Roche), and for the isolate, the QIAamp Viral RNA kit (Qiagen catalog 52906). All next-generation sequencing (NGS) libraries were prepared as previously described and used a probe enrichment modification of the TruSeq DNA Nano LP kit (catalog 20016328 Illumina) (4). Libraries were paired-end sequenced on an Illumina MiSeq with a v3 reagent kit (catalog MS-102–3003). Tools were run with default parameters unless otherwise specified. Read lengths were approximately 300 bp. Using Geneious Prime v2022.1.1, sequence ends and low-quality bases were trimmed and mapped to reference sequence (GenBank MK513622) with medium sensitivity and mapping quality 30. For the original specimen and library, from 302,382 paired reads, 285,646 were mapped to reference. The consensus sequence was generated using Highest Quality for threshold and minimum coverage of 10. Coding-complete genome sequence (15,678 nucleotides) was obtained with a mean depth of 3,321× and a GC content of 46.8%.

A U to C mutation was identified at nucleotide 3,221, causing a change from a stop to a glutamine codon. The coverage at nucleotide 3,221 was 1,983×, and the frequency of the C mutation was 98.6%. After an additional 57 nucleotides, another stop codon was identified at nucleotides 3,278–3,280, which occurs before the start of the subsequent M gene and would result in the addition of 19 amino acids to the carboxy terminus of the viral P. The overlapping open reading frame (ORF) C and V are not affected by this mutation.

To confirm these results, the NGS was repeated, and an available nasopharyngeal swab specimen from the same patient collected at the same time as the original urine specimen was included. The original urine specimen was also inoculated onto Vero/hSLAM cells (5), and the replicative virus, which caused a visible cytopathic effect, was isolated. NGS of all three libraries yielded the same mutation at position 3,221. Additionally, targeted reverse transcriptase polymerase chain reaction (RT-PCR) amplification followed by Sanger sequencing confirmed the C at position 3,221.

The measles P is 507 amino acid long and is involved in the transcription/replication of the viral RNA genome. Its structure is mostly disordered, but there is a folded domain (XD) at the carboxy-terminus which binds to the disordered N-tail domain of the viral nucleocapsid protein (Fig. 1A and B) (6). The putative extended XD domain of the P was analyzed by Alphafold2 and compared to the wild-type XD domain (Fig.1B) (7, 8). The P extension appears to be disordered with no indication that it interferes with the binding of the N-tail or develops an ordered structure upon N-tail binding. PeptideCutter (9) identified several potential protease cleavage sites in the P extension (Fig. 1C). Interestingly, cleavage at the 507 trypsin site would restore the wild-type protein.

## Data Availability

The coding-complete genome of MVs/British Columbia.CAN/4.19 has been deposited in Genbank under accession number OQ096316, and the raw read data have been submitted to SRA under BioProject PRJNA1017431 (accession numbers SRR26064243 (original specimen), SRR26338335 (nasopharyngeal specimen), and SRR26338391 (MeV isolate).
